# TWEAK blockade decreases atherosclerotic lesion size and progression through suppression of STAT1 signaling in diabetic mice

**DOI:** 10.1038/srep46679

**Published:** 2017-04-27

**Authors:** Valvanera Fernández-Laso, Cristina Sastre, Nerea Méndez-Barbero, Jesús Egido, Jose L. Martín-Ventura, Carmen Gómez-Guerrero, Luis M. Blanco-Colio

**Affiliations:** 1 Vascular Research Lab. FIIS-Fundación Jiménez Díaz, Av. Reyes Católicos 2, 28040, Madrid, Spain; 2 Spanish Biomedical Research Centre in Cardiovascular Disease (CIBERCV), Madrid, Spain; 3 Spanish Biomedical Research Centre in Diabetes and Associated Metabolic Disorders (CIBERDEM), Madrid, Spain.

## Abstract

Tumor necrosis factor-like weak inducer of apoptosis (TWEAK/*Tnfsf12*) is a cytokine implicated in different steps associated with vascular remodeling. However, the role of TWEAK under hyperglycemic conditions is currently unknown. Using two different approaches, genetic deletion of *Tnfsf12* and treatment with a TWEAK blocking mAb, we have analyzed the effect of TWEAK inhibition on atherosclerotic plaque progression and stability in streptozotocin-induced diabetic *ApoE* deficient mice. Genetic inactivation of *Tnfsf12* reduced atherosclerosis extension and severity in diabetic *ApoE* deficient mice. *Tnfsf12* deficient mice display a more stable plaque phenotype characterized by lower lipid and macrophage content within atherosclerotic plaques. A similar phenotype was observed in diabetic mice treated with anti-TWEAK mAb. The proatherosclerotic effects of TWEAK were mediated, at least in part, by STAT1 activation and expression of proinflammatory target genes (CCL5, CXCL10 and ICAM-1), both in plaques of *ApoE* mice and in cultured vascular smooth muscle cells (VSMCs) under hyperglycemic conditions. Loss-of-function experiments demonstrated that TWEAK induces proinflammatory genes mRNA expression through its receptor Fn14 and STAT1 activation in cultured VSMCs. Overall, TWEAK blockade delay plaque progression and alter plaque composition in diabetic atherosclerotic mice. Therapies aimed to inhibit TWEAK expression and/or function could protect from diabetic vascular complications.

Diabetes Mellitus (DM) prevalence is increasing worldwide. Thus, over 170 million people suffer from DM, and this number is predicted to increase to 366 million people by 2030^1^,^2^. Complications affecting the vasculature are the major causes of morbidity and mortality among diabetic subjects^3^. The biological mechanisms implicated in the development of diabetic vascular complications remain to be fully understood, but considerable attention has been paid to the role of endothelial dysfunction, inflammation and oxidative stress^4^,^5^. One molecule implicated in all of these processes is the tumor necrosis factor-like weak inducer of apoptosis (TWEAK, *Tnfsf12*)^6^. TWEAK is a cytokine belonging to the TNF superfamily that induces several physiological and pathological processes depending of the cell type and environment^7^. The sole functional receptor for TWEAK is fibroblast growth factor-inducible gene family that codes for a 14 kDa protein (Fn14)^8^. Fn14 expression is induced by oxidized-low density lipoproteins (ox-LDL) in cultured macrophages^9^ and by several molecules such as EGF, IL-1β, IFNγ, angiotensin II and thrombin in vascular smooth muscle cells (VSMCs)^7^,^10^. In the arterial wall, TWEAK is expressed in both healthy arteries and atherosclerotic plaques^10^. However, Fn14 expression is low or absent in normal arteries but is highly upregulated under pathological conditions including atherosclerotic plaques and abdominal aortic aneurysm of both, murine and human origin^10^,^11^,^12^,^13^. TWEAK is involved in different steps associated with vascular remodeling such as endothelial dysfunction, inflammation, proliferation and migration of VSMCs, angiogenesis and thrombosis^14^,^15^,^16^. In fact, TWEAK enhances vascular lesions associated with hyperlipidemia in apolipoprotein E knockout (*ApoE*^−/−^) mice by favouring an inflammatory response^11^. In addition, experimental studies have demonstrated that *Tnfsf12* deficient hypercholesterolemic mice, as well as mice treated with anti-TWEAK or Fn14-Fc display reduced atherosclerotic lesion size and increased plaque stability^12^,^13^.

TWEAK/Fn14 axis induces different signaling pathways such as nuclear factor -kappa B (NF-κB) and mitogen-activated protein kinases (MAPK), upregulating several cytokines implicated in the recruitment of inflammatory cells to the injured vessel wall^6^. In addition, TWEAK can also modulate Janus kinase/Signal transducers and activators of transcription (JAK/STAT) activation in tumor cells^17^. JAK/STAT pathway is a critical inflammatory mechanism by which hyperglycemia contribute to the pathogenesis of diabetes and its complications^18^. STAT responsive inflammatory genes include cytokines, chemokines and vasoactive proteins, many of them upregulated by diabetic conditions^19^,^20^.

However, despite these studies, the direct involvement of TWEAK/Fn14 axis in the development of vascular complications associated to diabetes is a still unexplored scenario. To investigate the role of TWEAK/Fn14 axis on diabetic-accelerated atherosclerosis, we studied the development of atherosclerotic plaques in *Tnfsf12*^+/+^*ApoE*^−/−^, *Tnfsf12*^−/−^*ApoE*^−/−^, and anti-TWEAK- or IgG-treated *ApoE*^−/−^ in streptozotocin-induced diabetic *ApoE* deficient mice. In addition, we analyzed the effect of TWEAK/Fn14 axis on JAK/STAT signaling both *in vitro* and *in vivo*.

## Results

### *Tnfsf12* deficiency reduces atherosclerotic burden and plaque size in diabetic ApoE knockout mice

**Figure 1:**
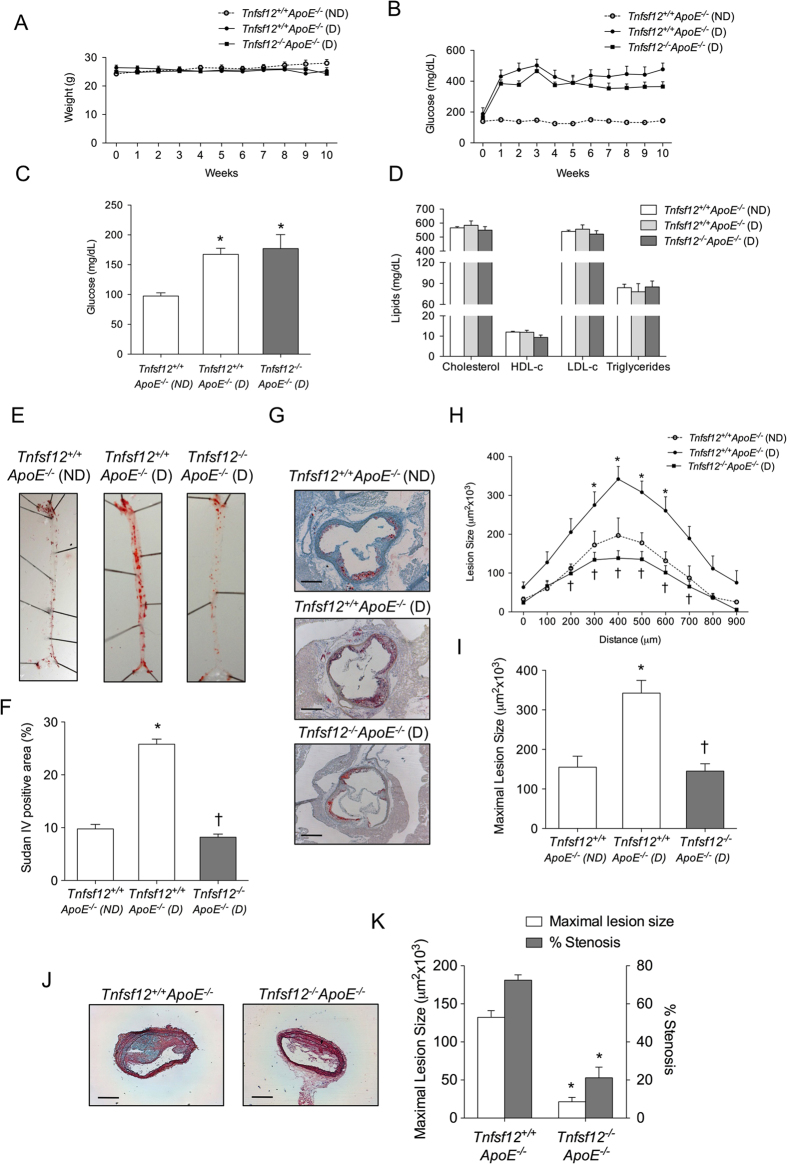
*Tnfsf12* deficiency reduces vascular damage in diabetic ApoE KO mice. (**A**) Averages body weight, (**B**) blood glucose levels (mean ± SEM) in the experimental groups of non-diabetic (ND) mice (*Tnfsf12*^+/+^*ApoE*^−/−^, N = 7) and diabetic (D) mice (*Tnfsf12*^+/+^*ApoE*^−/−^, N = 12; *Tnfsf12*^−/−^*ApoE*^−/−^, N = 12) during the 10 weeks of study. (**C**) Fasting blood glucose levels and (**D**) lipid measurements in the three groups at the end of the study. (**E**) Representative pinned-out en face aorta preparations and (**F**) quantification of atherosclerosis from diabetic mice stained with Sudan IV. Values shown are mean ± SD (N = 5 per group). *p < 0.001 *vs Tnfsf12*^+/+^*ApoE*^−/−^ (ND). ^†^p < 0.001 *vs Tnfsf12*^+/+^*ApoE*^−/−^ (**D**). (**G**) Representative Oil-Red-O/Hematoxylin staining and (**H**) Quantification of lesion area along the aortic root in diabetic and non-diabetic mice. Values shown are mean ± SD of *Tnfsf12*^+/+^*ApoE*^−/−^ (ND; N = 7), *Tnfsf12*^+/+^*ApoE*^−/−^ ((**D**) N = 12) and *Tnfsf12*^−/−^*ApoE*^−/−^ ((**D**) N = 12) mice. *p < 0.05 *vs Tnfsf12*^+/+^*ApoE*^−/−^ (ND). ^†^p < 0.01 *vs Tnfsf12*^+/+^*ApoE*^−/−^ (**D**). Scale bar, 200 μm. (**I**) Average of maximal lesions per group in the aortic root of mice. Values shown are mean ± SD of *Tnfsf12*^+/+^*ApoE*^−/−^ (ND; N = 7); *Tnfsf12*^+/+^*ApoE*^−/−^ ((**D**) N = 12) and *Tnfsf12*^−/−^*ApoE*^−/−^ ((**D**) N = 12) mice. *p < 0.001 *vs Tnfsf12*^+/+^*ApoE*^−/−^ (ND). ^†^p < 0.001 *vs Tnfsf12*^+/+^*ApoE*^−/−^ (**D**). (**J**) Representative photographs of Movat’s pentachrome sections of the brachiocephalic artery from different groups and (**K**) average of maximal lesion area and percentage of luminal occlusion in the brachiocephalic artery. Values shown are mean ± SD of *Tnfsf12*^+/+^*ApoE*^−/−^ ((**D**) N = 12) and *Tnfsf12*^−/−^*ApoE*^−/−^ ((**D**) N = 12) mice. *p < 0.001 *vs Tnfsf12*^+/+^*ApoE*^−/−^.

To analyze the effect of TWEAK deletion on diabetes mellitus-driven atherosclerosis, *Tnfsf12*^−/−^*ApoE*^−/−^ and their littermate *Tnfsf12*^+/+^*ApoE*^−/−^ mice (aged 16 weeks) were made diabetic by streptozotocin injection^21^. No statistically significant differences were observed in body weights (Fig. 1A) and glucose concentrations (Fig. 1B) between the two genotypes during the follow up. Additionally, there were no significant inter-group differences in the level of fasting glucose, cholesterol, HDL-c, LDL-c or triglycerides concentrations at the end of follow up (aged 26 weeks) (Fig. 1C,D). As expected, diabetic mice showed increased glucose concentrations (Fig. 1B,C) when comparing with non-diabetic *Tnfsf12*^+/+^*ApoE*^−/−^. However, no statistically significant differences were observed in body weights and lipid concentrations (Fig. 1A,D) between diabetic (D) or non-diabetic (ND) mice.

En face analysis of Sudan IV stained aorta showed a 67% reduction of lesion area in *Tnfsf12*^−/−^*ApoE*^−/−^ diabetic mice compared with *Tnfsf12*^+/+^*ApoE*^−/−^ diabetic mice (Fig. 1E,F; p < 0.05). Diabetic *Tnfsf12*^+/+^*ApoE*^−/−^ mice showed a 164% increase of lesion area compared with non-diabetic *Tnfsf12*^+/+^*ApoE*^−/−^ mice (Fig 1E,F). No differences were observed in lesion area between diabetic *Tnfsf12*^−/−^*ApoE*^−/−^ and non-diabetic *Tnfsf12*^+/+^*ApoE*^−/−^ (Fig 1E,F). Since aortic root and brachiocephalic artery are particularly prone to atherosclerosis, we also compared atherosclerotic lesion size in these regions. *Tnfsf12*^−/−^*ApoE*^−/−^ diabetic mice exhibited significantly reduced plaque size at the aortic root compared with *Tnfsf12*^+/+^*ApoE*^−/−^ diabetic mice (57% reduction; mean maximal lesion area; p < 0.001; Fig. 1G–I). Non-diabetic *Tnfsf12*^+/+^*ApoE*^−/−^ mice also showed a significantly reduced plaque size at the aortic root compared with *Tnfsf12*^+/+^*ApoE*^−/−^ diabetic mice (54% reduction; mean maximal lesion area; p < 0.001; Fig. 1G–I). No differences were observed between non-diabetic *Tnfsf12*^+/+^*ApoE*^−/−^ and diabetic *Tnfsf12*^−/−^*ApoE*^−/−^ (Fig. 1G–I). Atherosclerotic lesion size and luminal occlusion were also reduced in brachiocephalic artery of diabetic *Tnfsf12*^−/−^*ApoE*^−/−^ compared with diabetic *Tnfsf12*^+/+^*ApoE*^−/−^ control mice (84% and 71%; respectively) (Fig. 1J,K). Non-diabetic *Tnfsf12*^+/+^*ApoE*^−/−^ mice did not develop atherosclerotic plaques in their brachiocephalic artery at 26 weeks of age (not shown).

### *Tnfsf12* deletion diminishes the plaque progression in diabetic ApoE knockout mice

**Figure 2:**
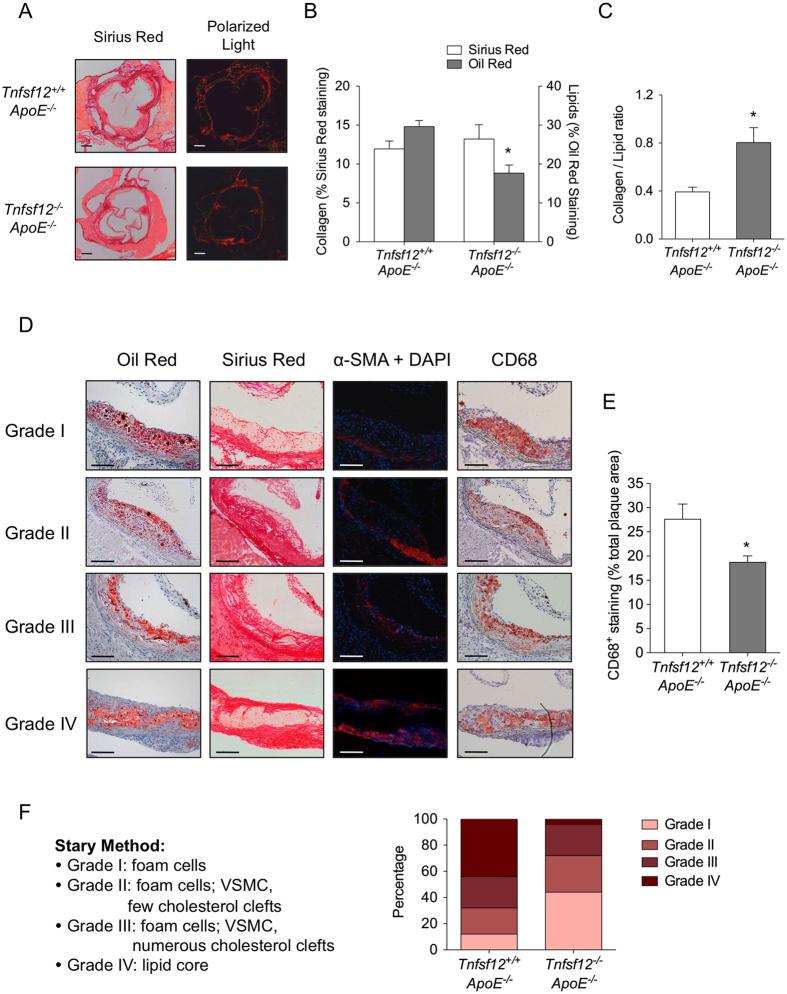
*Tnfsf12* deficiency reduces plaque progression in diabetic ApoE KO mice. (**A**) Representative images under bright field and polarized light of collagen staining with of Sirius Red and (**B**) quantification of Sirius red and Oil-Red-O in the aortic root of diabetic mice. Values shown are mean ± SD of *Tnfsf12*^+/+^*ApoE*^−/−^ (N = 12) and *Tnfsf12*^−/−^*ApoE*^−/−^ (N = 12) mice. *p < 0.001 *vs Tnfsf12*^+/+^*ApoE*^−/−^. Scale bar, 200 μm. (**C**) Plaque stability was assessed by collagen/lipid ratio. *p < 0.01 *vs Tnfsf12*^+/+^*ApoE*^−/−^. (**D**) Representative photographs of Oil-Red-O, Sirius red, α-SMA + DAPI and CD68 stained sections classified according to the Stary method (grade I to IV). Scale bar, 100 μm. (**E**) quantification of α-SMA and (**F**) CD68 in aortic root from diabetic mice from different groups. Values shown are mean ± SD of *Tnfsf12*^+/+^*ApoE*^−/−^ (N = 12) and *Tnfsf12*^−/−^*ApoE*^−/−^ (N = 12) mice. *p < 0.05 *vs Tnfsf12*^+/+^*ApoE*^−/−^. (**G**) Percentage of each grade from Stary method between diabetic groups. N = 25 plaques per group.

It has been described that the lipid deposits make the plaques more prone to rupture, while the collagen fibers stabilize them^22^. Lipid content, determined by Oil-Red-O staining, was 40% lower in *Tnfsf12*^−/−^*ApoE*^−/−^ compared to *Tnfsf12*^+/+^*ApoE*^−/−^ mice (Figs 1G and 2B; p < 0.001). However, collagen content, determined by Sirius Red staining, was similar in both genotypes (Fig. 2A,B). As a consequence, collagen/lipid ratio (a stability score) was significantly increased in *Tnfsf12*^−/−^*ApoE*^−/−^ compared to *Tnfsf12*^+/+^*ApoE*^−/−^ mice (Fig. 2C).

**Figure 3:**
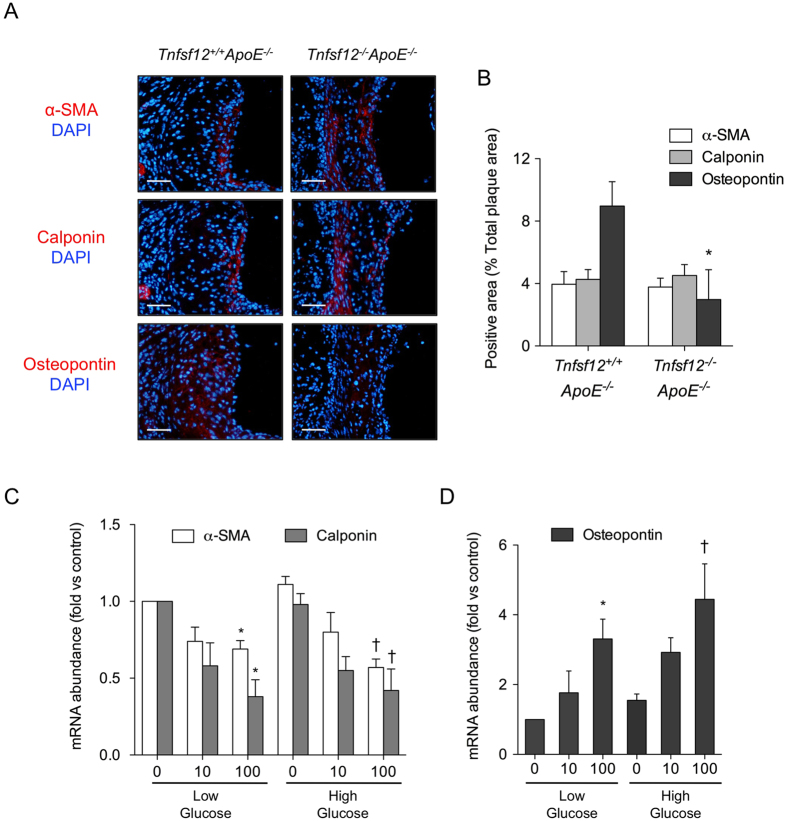
TWEAK induces VSMCs phenotypic switching. (**A**) Representative photographs and (**B**) quantification of contractile proteins (α-actin and calponin) and synthetic protein (osteopontin) in aortic lesions from *Tnfsf12*^+/+^*ApoE*^−/−^ and *Tnfsf12*^−/−^*ApoE*^−/−^ *diabetic* mice. Nuclei were counterstained with DAPI. Values shown are mean ± SD of *Tnfsf12*^+/+^*ApoE*^−/−^ (N = 10) and *Tnfsf12*^−/−^*ApoE*^−/−^ (N = 10) mice. *p < 0.001 vs *Tnfsf12*^+/+^*ApoE*^−/−^. Scale bar, 50 μm. (**C**) Quantitative real time PCR analysis of α-actin, calponin and (**D**) osteopontin mRNA expression in murine VSMCs stimulated with TWEAK, in the presence of low and high glucose concentrations. Values shown are mean ± SD. N = 4 experiments. *p < 0.05 *vs* low glucose without TWEAK. ^†^p < 0.05 vs high glucose without TWEAK.

Since inflammation plays a key role in atherosclerotic plaque stability, we also determined the presence of macrophages in the aortic root in both *Tnfsf12*^+/+^*ApoE*^−/−^ and *Tnfsf12*^−/−^*ApoE*^−/−^ diabetic mice. The content of CD68^+^ cells was reduced by 32% (p < 0.05) in *Tnfsf12*^−/−^*ApoE*^−/−^ atherosclerotic plaques compared with *Tnfsf12*^+/+^*ApoE*^−/−^ (Fig. 2D,E). In addition, we also analyzed the phenotypic characteristics of VSMCs by studying mRNA expression of contractile (α-actin and calponin) and secretory (osteopontin) markers in the aortic root in both *Tnfsf12*^+/+^*ApoE*^−/−^ and *Tnfsf12*^−/−^*ApoE*^−/−^ diabetic mice. The content of osteopontin^+^ VSMCs was reduced in *Tnfsf12*^−/−^*ApoE*^−/−^ atherosclerotic plaques compared with *Tnfsf12*^+/+^*ApoE*^−/−^ (Fig. 3A,B). No changes were observed in the content of contractile markers α-actin or calponin in atherosclerotic plaques from *Tnfsf12*^+/+^*ApoE*^−/−^ and *Tnfsf12*^−/−^*ApoE*^−/−^ diabetic mice (Fig. 3A,B). In order to confirm the role of TWEAK on VSMCs phenotypic switching, contractile and secretory VSMCs markers were analyzed in cultured murine VSMCs. TWEAK (0.1 μg/mL) increased osteopontin and decreased α-actin and calponin mRNA expression in cultured VSMCs (Fig. 3C), indicating that TWEAK favors a switching toward a secretory VSMCs phenotype *in vitro*. TWEAK treatment did not induce VSMCs apoptosis in our experimental conditions (not shown).

To analyze the effect of TWEAK deficiency on the progression of the atherosclerotic plaques in diabetic mice, we used the Stary method, which classified the atherosclerotic lesions based on their histological composition and structure^23^: grade I, early plaques containing only macrophages; grade II, lesions containing macrophages, VSMCs and a few cholesterol clefts; grade III, lesions containing macrophages, VSMCs and numerous cholesterol clefts; and grade IV, advanced plaques containing macrophages, VSMCs and a large lipid core (Fig. 2D). After 10 weeks of diabetes induction, ≈40% plaques in *Tnfsf12*^+/+^*ApoE*^−/−^ were advanced plaques (grade IV) while only ≈10% plaques were early lesions (grade I) (Fig. 2F). In contrast, the percentage of advanced plaques was only ≈4% in *Tnfsf12*^−/−^*ApoE*^−/−^ while ≈40% plaques were early plaques (Fig. 2F). These results therefore indicate that TWEAK plays a key role in diabetic-accelerated atherosclerosis progression.

### *Tnfsf12* deletion reduces STAT1 proinflammatory response under hyperglycemic conditions

**Figure 4:**
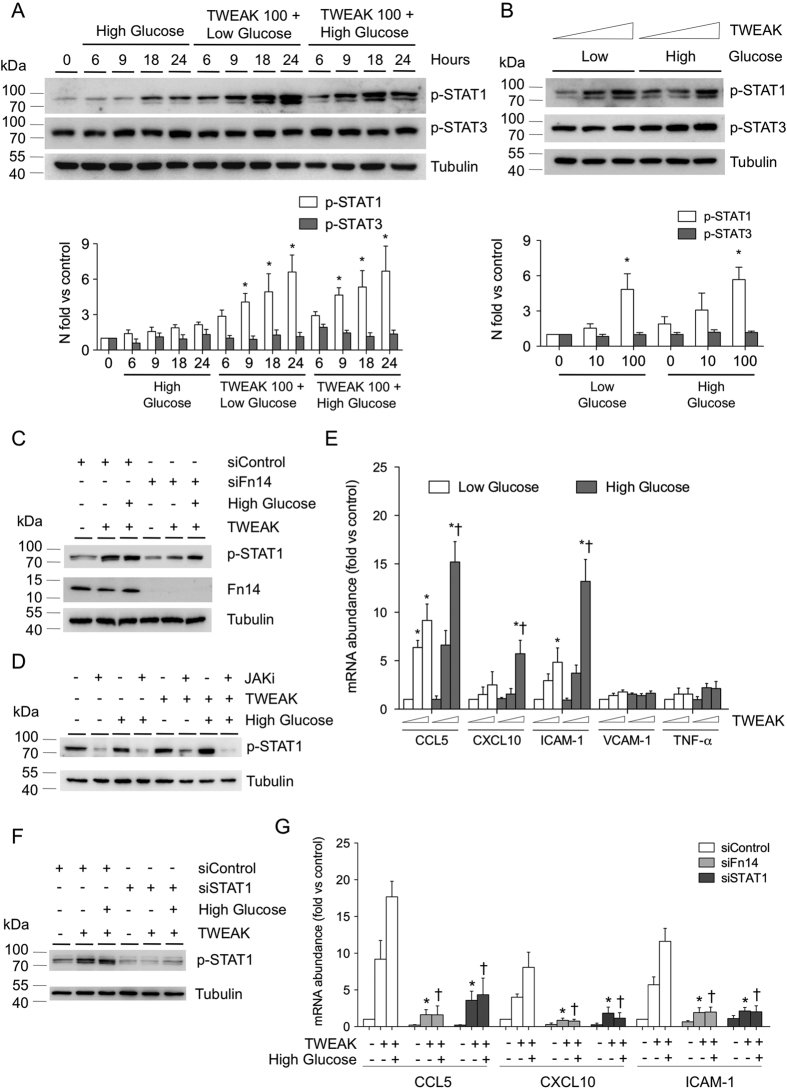
TWEAK activates STAT1 and induces pro-inflammatory chemokine expression in VSMCs. (**A**) Representative western-blot analysis of pSTAT1 and pSTAT3 in murine VSMCs incubated with low (5.5 mmol/L) or high (35 mmol/L) glucose and rTWEAK (100 ng/mL) for 0–24 hours. Values normalized to tubulin expression are expressed as multiples of control conditions (arbitrarily set to 1). N = 4 independent experiments. Full-length blots are in Supplemental Figure S1. (**B**) Representative western-blot analysis of pSTAT1 and pSTAT3 in murine VSMCs incubated with low (5.5 mmol/L) or high (35 mmol/L) glucose and rTWEAK (0, 10 or 100 ng/mL) for 24 hours. Values normalized to tubulin expression are expressed as multiples of control conditions (arbitrarily set to 1). N = 4 independent experiments. Full-length blots are in Supplemental Figure S2. (**C**) Immunodetection of p-STAT1 and Fn14 in murine VSMCs under transfection transfected with either specific siRNA for Fn14 or universal negative control siRNA and stimulated with rTWEAK (100 ng/mL) for 24 hours. N = 4 independent experiments. Full-length blots are in Supplemental Figure S3. (**D**) Representative western-blot analysis of pSTAT1 in murine VSMCs incubated with low (5.5 mmol/L) or high (35 mmol/L) glucose and rTWEAK (100 ng/mL) for 24 hours in presence or absence of JAK inhibitor (250 nM). N = 4 independent experiments. Full-length blots are in Supplemental Figure S4. (**E**) Quantitative real time PCR analysis of CCL5, CXCL10, ICAM-1, VCAM-1 and TNF-α mRNA expression in murine VSMCs stimulated with TWEAK, in the presence of low or high glucose concentrations. Values shown are mean ± SD. N = 6 experiments *p < 0.05 *vs* control. (**F**) Representative western-blot analysis of p-STAT1 in murine VSMCs transfected with control or STAT1 siRNA. N = 3 independent experiments. Full-length blots are in Supplemental Figure S4. (**G**) Quantitative real-time PCR analysis of the indicated genes in murine VSMCs stimulated with TWEAK (100 ng/mL) for 24 hours, in the presence low (5.5 mmol/L) or high (35 mmol/L) glucose concentrations after transfection with specific siRNA for Fn14, STAT1, or siRNA negative control. Values shown are mean ± SD. N = 5 independent experiments *p < 0.05 *vs* TWEAK, low glucose and siRNA control. ^†^p < 0.05 *vs* TWEAK, high glucose and siRNA control.

JAK/STAT is an essential intracellular pathway of cytokines and inflammatory factors that regulates key atherosclerotic processes under diabetic conditions^20^. To analyze the role of TWEAK on JAK/STAT activation, murine VSMCs were cultured in the presence of low or high glucose concentrations and coincubated in the presence or absence of murine recombinant TWEAK (0.01–0.1 μg/mL). TWEAK induced in a dose- and time-dependent manner the phosphorylation of STAT1, but not STAT3, in VSMCs under both low-glucose and high-glucose conditions (Fig. 4A,B). No differences were observed in STAT-1 phosphorylation when TWEAK was incubated in the presence of D-glucose or L-Glucose (not shown). In loss-of-function experiments using siRNA against Fn14, TWEAK is unable to induce STAT1 phosphorylation in VSMCs (Fig. 4C), indicating that TWEAK activates STAT1 through its functional receptor. In addition, use of specific JAK inhibitor abolished the TWEAK-induced STAT1 phophorylation in these cells (Fig. 4D). These data indicate that TWEAK mediated STAT1 phosphorylation acts through the canonical JAK-STAT signaling pathway in VSMCs.

We analyzed the mRNA expression of proinflammatory STAT-regulated genes including CCL5, CXCL10 and ICAM-1 in cultured VSMCs. TWEAK increased CCL5 and ICAM-1 mRNA expression in VSMCs cultured under low-glucose concentrations (Fig. 4E). In addition, TWEAK also augmented CCL5, CXCL10 and ICAM-1 mRNA expression in a hyperglycemic scenario (Fig. 4E). Interestingly, the effect of TWEAK on CCL5 and ICAM-1 expression was superior in high *vs* low glucose concentrations, indicating an additive effect between TWEAK and glucose on the induction of inflammatory genes. No statistical significant changes were observed in TNFα or VCAM-1 mRNA expression (Fig. 4E).

Loss-of-function experiments using siRNA against Fn14 or STAT1 demonstrate that TWEAK induce CCL5, CXCL10 and ICAM-1 mRNA expression through its sole receptor Fn14 and further STAT1 activation in VSMCs (Fig. 4C,F and G).

**Figure 5:**
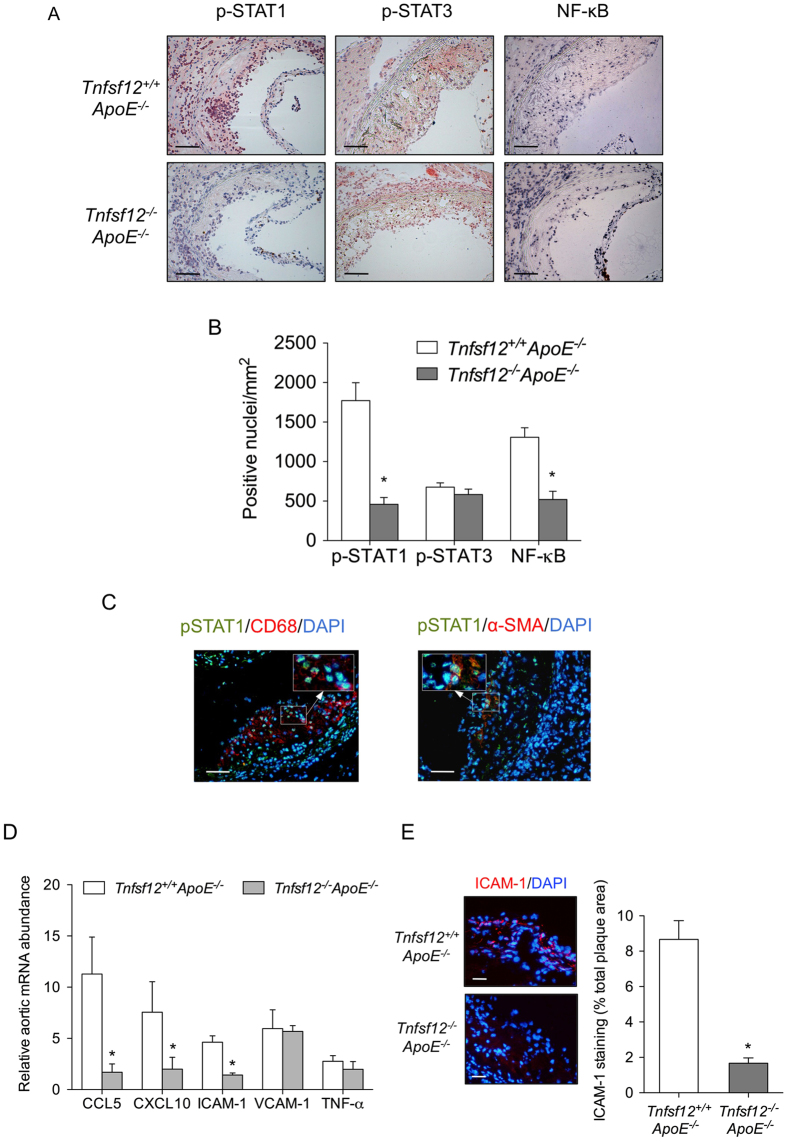
TWEAK activates STAT1 and induces pro-inflammatory chemokine expression in diabetic mice. (**A**) Representative immunostaining and (**B**) quantification of p-STAT1, p-STAT3 and activated NF-κB in aortic lesions from *Tnfsf12*^+/+^*ApoE*^−/−^ and *Tnfsf12*^−/−^*ApoE*^−/−^ diabetic mice. Values shown are mean ± SD of *Tnfsf12*^+/+^*ApoE*^−/−^ (N = 12) and *Tnfsf12*^−/−^*ApoE*^−/−^ (N = 12) mice. Scale bar, 200 μm. *p < 0.001 vs *Tnfsf12*^+/+^*ApoE*^−/−^. (**C**) Low- and high-magnification views of double immunostaining for p-STAT1^+^ cells (green) and CD68^+^ or α-SMA^+^ cells (red) in atherosclerotic plaques present in the aortic root of mice. Nuclei were counterstained with DAPI. Scale bar, 50 μm. (**D**) Quantitative real time PCR analysis on CCL5, CXCL10, ICAM-1, VCAM-1 and TNF-α mRNA expression in total aorta of *Tnfsf12*^+/+^*ApoE*^−/−^ and *Tnfsf12*^+/+^*ApoE*^−/−^ mice. Values shown are mean ± SD of 7 animals included in each group. *p < 0.05 vs *Tnfsf12*^+/+^*ApoE*^−/−^. (**E**) Representative immunostaining and quantification of ICAM-1 in aortic lesions from *Tnfsf12*^+/+^*ApoE*^−/−^ and *Tnfsf12*^−/−^*ApoE*^−/−^ diabetic mice. Values shown are mean ± SD of *Tnfsf12*^+/+^*ApoE*^−/−^ (N = 10) and *Tnfsf12*^−/−^*ApoE*^−/−^ (N = 10) mice. *p < 0.001 vs *Tnfsf12*^+/+^*ApoE*^−/−^. Scale bar, 25 μm.

To confirm the *in vitro* results in our animal model, we measured the content of p-STAT1 and p-STAT3 in the aortic plaques from both groups, *Tnfsf12*^+/+^*ApoE*^−/−^ and *Tnfsf12*^−/−^*ApoE*^−/−^. We observed a significant reduction of pSTAT1 immunostaining in the atherosclerotic plaques of *Tnfsf*12^−/−^*ApoE*^−/−^ compared with those of controls (Fig. 5A,B). No significant changes were found in pSTAT3 levels (Fig. 5A,B). Double immunofluorescence studies showed that pSTAT1 colocalized with both CD68^+^ and α-SMA^+^ cells within atherosclerotic lesions (Fig. 5C). In addition, we analyzed the activation of NF-κB, a key transcription factor involved in the inflammatory response associated to atherosclerotic plaque development. We observed a significant reduction of NF-κB activation in the atherosclerotic plaques of diabetic *Tnfsf*12^−/−^*ApoE*^−/−^ compared with those of diabetic controls (Fig. 5A,B).

We next investigated the expression of the STAT1 downstream genes in aortic extract from both *Tnfsf12*^+/+^*ApoE*^−/−^ and *Tnfsf12*^−/−^*ApoE*^−/−^. Accordingly, the mRNA expression levels of CCL5, CXCL10 and ICAM-1 were reduced in *Tnfsf12*^−/−^*ApoE*^−/−^ compared with control mice (Fig. 5D). No statistical significant changes were observed in TNFα or VCAM-1 mRNA expression (Fig. 5D). To confirm the effect of TWEAK deletion on proinflammatory genes expression *in vivo*, we analyzed ICAM-1 protein expression in the aortic root of different mice groups included in our study. We observed a significant reduction in ICAM-1 protein expression in *Tnfsf*12^−/−^*ApoE*^−/−^ compared with those of diabetic controls (Fig. 5E).

### Anti-TWEAK mAb treatment reduces atherosclerotic burden, plaque size and progression in diabetic *Tnfsf12* ^+/+^ *ApoE* ^−/−^

**Figure 6:**
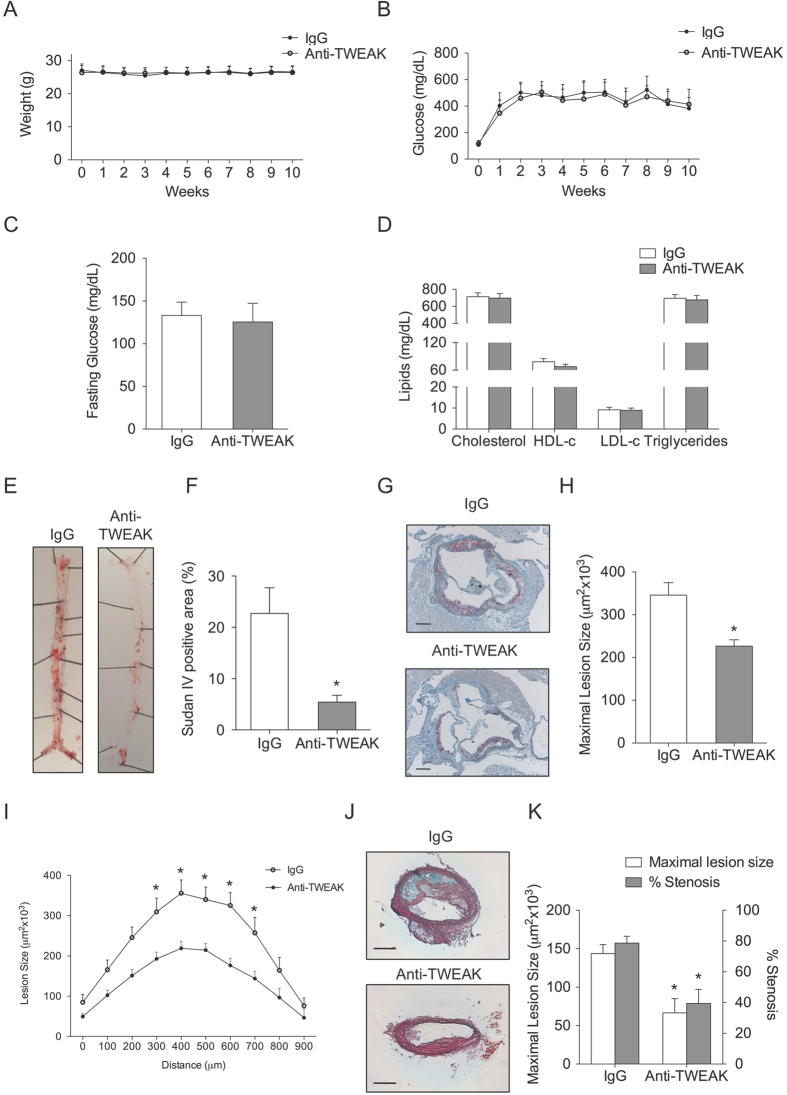
Anti-TWEAK mAb treatment reduces vascular damage in diabetic ApoE KO. (**A**) Average weight and (**B**) blood glucose levels (mean ± SEM) of IgG (N = 13) and anti-TWEAK (N = 13) mice during the 10 weeks of study. (**C**) Fasting blood glucose levels and (**D**) lipid measurements in both groups at the end of the study. (**E**) Representative pinned-out en face aorta preparations and (**F**) quantification of atherosclerosis from diabetic mice stained with Sudan-IV. Values shown are mean ± SD (N = 5 per group). *p < 0.01 *vs* IgG. (**G**) Representative images of Oil-Red-O/Hematoxylin staining in the arotic root of diabetic mice and (**H**) average of maximal lesions per group and quantification of Oil-Red-O staining in the aortic root of mice. Values shown are mean ± SD of IgG (N = 13) and anti-TWEAK (N = 13) mice. *p < 0.005 *vs* IgG. Scale bar, 200 μm. (**I**) Quantification of lesion area along the aortic root of diabetic mice treated with IgG or Anti-TWEAK. Values shown are mean ± SD of IgG and anti-TWEAK (N = 13 in each group) mice. *p < 0.01 *vs* IgG. (**J**) Representative photographs of Movat’s pentachrome staining in brachiocephalic artery sections from different groups. (**K**) Average of maximal lesion area per group and percentage of luminal occlusion in the brachiocephalic artery. Values shown are mean ± SD of IgG and anti-TWEAK (N = 13 in each group) mice. *p < 0.005 *vs* IgG.

We next investigate whether TWEAK related-effects can be modulated by therapeutic intervention with anti-TWEAK mAb. For that purpose, *Tnfsf12*^+/+^*ApoE*^−/−^ mice (aged 16 weeks) were made diabetic by streptozotocin injection. After that, animals were randomized to receive anti-TWEAK mAb or IgG (10 mg/kg/twice a week) during 10 weeks. No statistically significant differences were observed in body weights (Fig. 6A) and glucose concentrations (Fig. 6B) between the two groups during the follow up. No statistically significant inter-group differences were observed in fasting glucose, cholesterol, HDL-c, LDL-c or triglycerides concentrations at the end of follow up (Fig. 6C,D).

En face analysis of Sudan IV stained aorta revealed that aortic lesion area in animals treated with anti-TWEAK was reduced in a 76% compared with control mice (Fig. 6E,F; p < 0.01). In addition, mice treated with anti-TWEAK exhibited a diminished plaque size at the level of the aortic root compared with IgG-treated mice (p < 0.005) (Fig. 6G–I). Moreover, mice treated with anti-TWEAK also showed a reduction in lesion size and luminal occlusion (54% and 23%; respectively; p < 0.005 for both) in plaques from brachiocephalic artery compared with IgG-treated mice (Fig. 6J,K).

**Figure 7:**
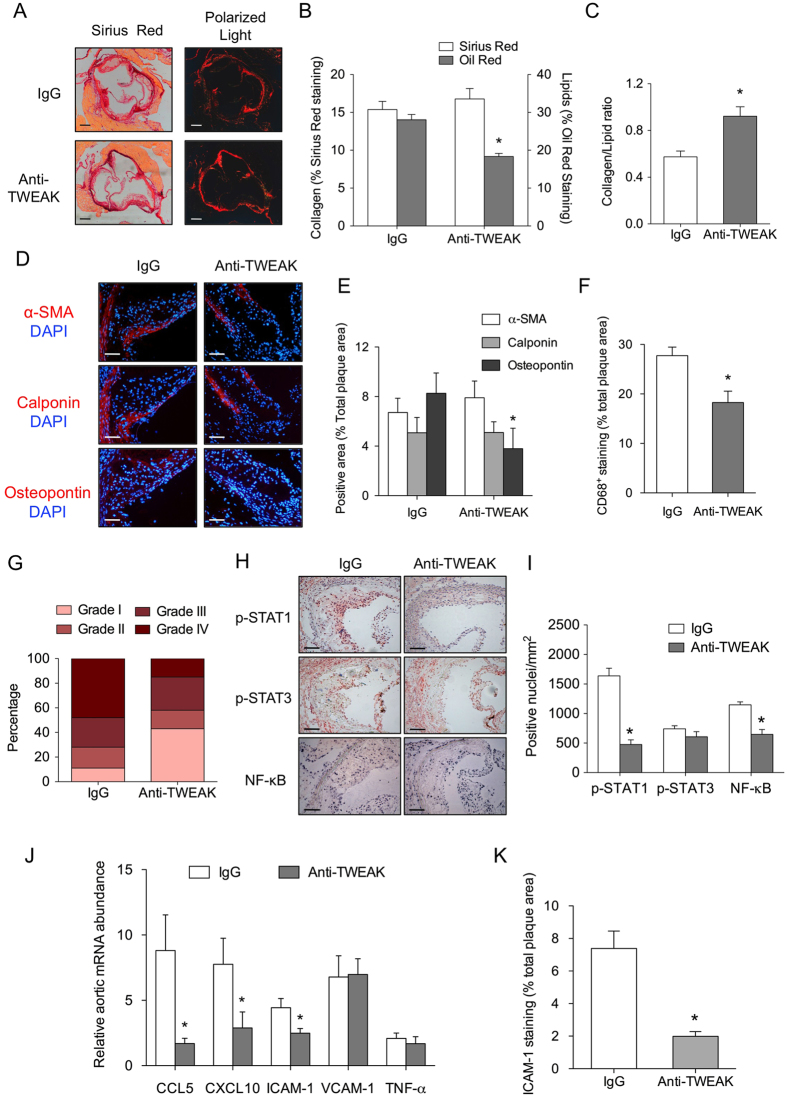
Anti-TWEAK mAb treatment diminished plaque progression and STAT1 activation in diabetic mice. (**A**) Representative images (bright field and polarized light) of Sirius Red staining and (**B**) quantification of Sirius red and Oil-Red-O in the aortic root of diabetic mice treated with anti-TWEAK or IgG treated (N = 13 in each group) mice. Values shown are mean ± SD. *p < 0.05 *vs* IgG. Scale bar, 200 μm. (**C**) Plaque stability was assessed by collagen/lipid ratio. *p < 0.01 *vs* IgG. (**D**) Representative photographs and (**E**) quantification of contractile proteins (α-actin and calponin) and synthetic protein (osteopontin) in the aortic root of diabetic mice treated with anti-TWEAK or IgG treated (N = 10 in each group) mice. Nuclei were counterstained with DAPI. Values shown are mean ± SD. *p < 0.05 vs IgG. Scale bar, 50 μm. (**F**) Quantification of CD68 in aortic root from both groups. Values shown are mean ± SD of IgG and anti-TWEAK (N = 13 in each group) mice. *p < 0.005 *vs* IgG. (**G**) Percentage of each grade from Stary method in both groups. N = 29 and N = 33 plaques for IgG and anti-TWEAK group, respectively. (**H**) Representative immunostaining and (**I**) quantification of p-STAT1, p-STAT3 and activated NF-kB in atherosclerotic plaques in aortic root from mice treated with anti-TWEAK or IgG. Values shown are mean ± SD of IgG and anti-TWEAK (N = 13 in each group) mice. *p < 0.001 *vs* IgG. (**J**) Quantitative real time PCR analysis of CCL5, CXCL10, ICAM-1, VCAM-1 and TNF-α mRNA expression in total aorta of mice treated with anti-TWEAK or IgG. Values shown are mean ± SD of 7 animals included in each group. *p < 0.05 *vs* IgG. (**K**) Quantification of ICAM-1 in atherosclerotic plaques present in the aortic root of anti-TWEAK or IgG treated mice (N = 10 per group). Nuclei were counterstained with DAPI. *p < 0.001 *vs* IgG.

To analyze plaque composition, we evaluated the content of collagen, lipids, VSMCs and macrophages in both, anti-TWEAK and IgG-treated mice. Lipid content was 35% lower in mice treated with anti-TWEAK compared to IgG-treated mice (Figs 6G and 7B; p < 0.001), while no statistically differences were observed in collagen content (Fig. 7A,B). Plaques of anti-TWEAK treated mice showed significant increased collagen/lipid ratio compared to IgG-treated mice (Fig. 7C). The content of osteopontin^+^ VSMCs was also reduced in anti-TWEAK compared with IgG-treated mice (Fig. 7D,E). No changes were observed in the content of contractile markers of VSMCs α-actin or calponin in atherosclerotic plaques from anti-TWEAK and IgG diabetic mice (Fig. 7D,E). In addition, anti-TWEAK treatment reduced significantly macrophage content compared with IgG (27% reduction; p < 0.005) (Fig. 7F).

Consistent with data obtained from *Tnfsf12*^−/−^*ApoE*^−/−^ mice, ≈15% plaques were advanced lesions (grade IV) while ≈40% plaques in anti-TWEAK-treated mice were early lesion (grade 1) (Fig. 7G). In contrast, the percentage of advanced plaques was ≈40% in IgG-treated mice while ≈10% plaques were early lesions (Fig. 7G). These data therefore indicate that anti-TWEAK treatment decreases diabetic-accelerated atherosclerosis progression.

Finally, STAT1 activation was significantly lower in plaques of mice treated with anti-TWEAK compared with those treated with IgG (Fig. 7H,I). No changes were observed in pSTAT3 content in atherosclerotic plaques of aortic roots from both treated groups. Anti-TWEAK treatment also diminished NF-κB activation in atherosclerotic plaques of mice compared with IgG-treated mice (Fig. 7H,I).

Total aorta mRNA expression of STAT1 downstream genes CCL5, CXCL10 and ICAM-1 were reduced in anti-TWEAK treated mice compare with those treated with IgG (Fig. 7J). In addition, ICAM-1 expression was also reduced in aortic root from anti-TWEAK treated mice compared with IgG group (Fig. 7K).

## Discussion

Atherosclerosis plaque development, progression, and rupture involve complex pathological mechanisms. The main focus of our study was to determine whether TWEAK/Fn14 axis mediates vascular inflammation in response to hyperglycemia. Previous studies have demonstrated that TWEAK is critically implicated in the development and progression of atherosclerotic plaques in hypercholesterolemic mice^9^,^11^,^12^. In addition, circulating levels of soluble TWEAK are associated with the presence of type 1 diabetes^24^. These studies suggest that hyperglycemia or other metabolic changes associated with diabetes may contribute to the development of diabetic vasculopathy through TWEAK-induced vascular inflammation. Our present findings support that TWEAK/Fn14 axis mediates inflammatory responses under hyperglycemic conditions. Indeed, *Tnfsf12* deletion or anti-TWEAK treatment diminished atherosclerotic lesion size in both aortic root and brachiocephalic arteries of diabetic *ApoE* KO mice. The absence of TWEAK was accompanied by reduction of lipid, macrophage and secretory VSMCs content, and diminishes of the STAT dependent proinflammatory genes in atherosclerotic tissue. Finally, TWEAK induced STAT1 activation and cytokines expression in cultured VSMCs. Overall, our data indicate that TWEAK inhibition diminished the inflammatory response and atherosclerotic plaque progression in diabetic mice.

It has been reported that TWEAK induces systemic metabolic complications of high fat diet-induced obesity in aged mice^25^. In addition, aged TWEAK-Tg mice (18 month-old) have visceral obesity, insulin resistance and metabolic dysfunction. In contrast, younger TWEAK-Tg mice (4 month-old) do not show any differences in body weight or glucose or insulin tolerance^26^. In the same line of evidence, we have not observed any differences in body weight, lipid profile or hyperglycemia between both *Tnfsf12*^+/+^*ApoE*^−/−^ and *Tnfsf12*^−/−^*ApoE*^−/−^ mice. Likewise, the beneficial effect of anti-TWEAK Ab was independent of any appreciable influence on the metabolic severity of diabetes mellitus, as evidenced by no changes in hyperglycemia, lipid profile, or body weight. These data indicate that the effects observed by *Tnfsf12* deletion or anti-TWEAK Ab in our experimental conditions are not related with metabolic changes.

TWEAK and Fn14 have been detected in both VSMCs and macrophages within human atherosclerotic plaques^10^,^27^ and their expression has been related with inflammatory responses and oxidative stress^11^,^28^,^29^. Recent reports demonstrate the role of TWEAK/Fn14 in different models of tissue remodeling after injury^30^. In fact, gain- or loss-of-function approaches have demonstrated that TWEAK/Fn14 axis participates in the development of atherosclerotic plaques and abdominal aortic aneurysms in mice^11^,^12^,^13^.

Here, we have observed that diabetic *Tnfsf12*^+/+^*ApoE*^−/−^ showed a 120% increase in lesion size of aortic root compared with non-diabetic *Tnfsf12*^+/+^*ApoE*^−/−^. *Tnfsf12* deficiency or anti-TWEAK treatment attenuates atherosclerotic lesion burden in terms of both, lesion size and severity in diabetic mice. While most lesions in *Tnfsf12*^+/+^*ApoE*^−/−^ or IgG treated mice were advanced (grade IV), *Tnfsf12*^−/−^*ApoE*^−/−^ or anti-TWEAK treated mice exhibited a higher number of early lesions (grade I). These data indicate that TWEAK deletion or blockade delayed atherosclerotic plaques progression in hyperlipidemic mice under hyperglycemic conditions.

Clinically, lesion composition rather than size or degree of stenosis of atherosclerotic plaques determines the likelihood of plaque rupture and subsequent thrombotic complications^31^. Although *ApoE* deficient mice are not particularly prone to develop unstable plaques in their aorta^32^, it was possible that *Tnfsf12* deficiency might decrease features of plaque vulnerability in diabetic mice. Unstable plaques are rich in lipids and inflammatory cells and exhibit a substantial loss of VSMCs and collagen content^31^. Lipid accumulation within arterial wall increases monocytes recruitment and subsequent differentiation to macrophage and foam cells. The presence of ox-LDL within atherosclerotic plaques increases proinflammatory molecules and matrix metalloproteinases expression by macrophages that contribute to plaque instability. In addition, VSMCs exhibit phenotypic and functional plasticity in order to respond to vascular injury. In case of vessel damage, VSMCs are able to switch from the quiescent ‘contractile’ phenotype to the ‘proinflammatory’ phenotype. It is known that ox-LDL and hyperglycemia promotes phenotypic switch in VSMCs towards the proinflammatory phenotype associated with their dedifferentiation, proliferation and migration^33^. Ox-LDL induces the expression of various proinflammatory cytokines, chemokines and adhesion molecules in VSMCs^34^. In addition, ox-LDL increases metalloproteinases activity in VSMCs^35^ that contributes to matrix degradation and plaque destabilization. In this sense, we observed that plaques from *Tnfsf12*^−/−^*ApoE*^−/−^ or anti-TWEAK treated diabetic mice exhibit reduced lipid, macrophage content and secretory VSMCs, without changes in contractile VSMCs or collagen content, in their atherosclerotic plaques. The increment of the number of secretory VSMCs could be related with an augmentation proliferation induced by TWEAK as previously it was demonstrated^36^.

As a consequence, increased score of plaque stability such as collagen/lipid ratio was observed in *Tnfsf12*^−/−^*ApoE*^−/−^ or anti-TWEAK treated diabetic mice. The diminution observed in lipid content could be related with a reduction of macrophage/foam cells as it was observed in *Tnfsf12*^−/−^*ApoE*^−/−^ hyperlipidemic mice^12^. Moreover, it has been demonstrated that Fn14-Fc treated mice have smaller macrophages within their atherosclerotic plaques, effect attributable to reduced lipid uptake^9^. These data, together with the lower macrophage content in plaques of *Tnfsf12* deficient mice suggest that *Tnfsf12* inactivation or blockade might increase plaque stability.

The underlying mechanism of the reduction of inflammatory cells namely macrophages by *Tnfsf12* deletion or anti-TWEAK treatment is related to the reduction in proinflammatory cytokines expression. Initially, we observed lower STAT1 phosphorylation in atherosclerotic plaques from both *Tnfsf12*^−/−^ and anti-TWEAK treated mice compared with their respective control mice. In addition, we have observed an increased STAT1 activation under hyperglycemic conditions or in the presence of TWEAK in cultured VSMCs. This activation was specific of STAT1 since other family member (STAT3), was not affected by glucose or TWEAK concentrations under our experimental conditions. TWEAK-mediated STAT1 phosphorylation was dependent on the presence of its unique functional receptor Fn14^8^. Thus, siRNA against Fn14 prevented STAT1 activation induced by TWEAK in VSMCs. These results are in agreement with previous data showing that TWEAK induced STAT1 activation in tumor cells^17^. In addition, a JAK inhibitor blocked STAT1 phosphorylation induced by TWEAK. Our data thus indicate that canonical JAK/STAT signaling pathway mediates TWEAK inflammatory responses in VSMCs. The slow kinetics of STAT1 phosphorylation in TWEAK-stimulated cells suggests that the induction of a soluble factor may be responsible for inducing STAT-1 activation. In fact, it has been demonstrated that neutralizing antibodies against IFN-β inhibited STAT1 phosphorylation induced by TWEAK in WiDr tumor cells^17^. JAK/STAT pathway plays an important role in atherosclerotic plaques development. In fact, STAT isoforms are expressed in the inflammatory regions of human atherosclerotic plaques and in experimental models of atherosclerosis^37^,^38^,^39^. In addition, total or cell-restricted STAT1 or STAT3 deficiency prevents atherosclerosis in mice^37^,^38^,^40^. JAK/STAT is also a key inflammatory mechanism by which hyperglycemia contributes to the diabetic complications^18^,^20^. Targeting JAK/STAT activation inhibits proinflammatory genes expression, leukocyte infiltration, and vascular cell activation, thereby preventing development and progression of atherosclerosis in diabetic mice^20^,^41^. The inflammatory response induced by JAK/STAT pathway comprises different genes including chemokines, cytokines, enzymes and vasoactive proteins, many of them upregulated by diabetic conditions^19^. Of these, we have observed that TWEAK induced CCL5 and ICAM-1 mRNA expression in cultured VSMCs. Hyperglycemic conditions elicited an additive effect on CCL5, CXCL10 and ICAM-1 mRNA expression in VSMCs under TWEAK stimulation. In addition, loss-of-function experiments using siRNA against STAT1 demonstrated that TWEAK induces CCL5, CXCL10 and ICAM-1 mRNA, at least in part, through STAT1 activation. The *in vitro* findings were confirmed *in vivo*. We have observed that activation of STAT1, but not STAT3, along with CCL5, CXCL10 and ICAM-1 mRNA expression were reduced in the whole aorta from *Tnfsf12* deficient or anti-TWEAK treated mice.

Although STAT1 activation seems to be important in the inflammatory response induced by TWEAK under hyperglycemic conditions, we cannot exclude that other potential mechanism/s could participate in the inflammatory response associated to atherosclerotic plaque development. In fact, it has been demonstrated that recombinant TWEAK injection increases NF-κB activation and chemokines (CCL2 and CCL5) expression in atherosclerotic plaques of hyperlipidemic mice^11^. In addition, *Tnfsf12* deletion or anti-TWEAK treatment diminished NF-κB activation and chemokines expression in normoglycemic *ApoE* deficient mice^12^. Now, we observed attenuated NF-κB activation in atheroma from *Tnfsf12* deficient and anti-TWEAK treated diabetic mice compared with control mice. In this context, it is possible that both pathways, JAK/STAT and NF-κB, participate in the inflammatory response induced by TWEAK under a hyperglycemic environment.

In conclusion, this is the first study to analyze the importance of TWEAK/Fn14 axis in experimental diabetes-associated atherosclerosis. Our experimental data provide evidence that TWEAK inhibition ameliorates diabetes-driven atherosclerosis through the attenuation of STAT1 activation and proinflammatory cytokines expression, thus resulting in reduced atherosclerotic lesion development and increased plaque stability. TWEAK blockade could be a novel therapeutic approach to limit diabetic vascular complications.

## Methods

### Diabetes Model

Animal studies were performed according to the Directive 2010/63/EU of the European Parliament and were approved by the Institutional Animal Care and Use Committee (FIIS-Fundación Jiménez Díaz). Experimental diabetes model of insulin deficiency was induced in 16-week-old male *Tnfsf12*^−/−^*ApoE*^−/−^ and their littermate control *Tnfsf12*^+/+^*ApoE*^−/−^ mice by two daily intraperitoneal injections of streptozotocin (125 mg/kg/day; Sigma-Aldrich, St. Louis, MO) (20). Animals maintained on standard diet were monitored every 2**–**3 days for body weight and nonfasting blood glucose. Severely hyperglycemic mice (blood glucose >29 mmol/L) received insulin (1**–**1.5 IU) to maintain blood glucose levels within a more tolerable range. Twenty-six *Tnfsf12*^+/+^*ApoE*^−/−^ mice with overt diabetes (glucose >19.4 mmol/L) were randomized to receive 10 weeks of treatment with anti-TWEAK mAb (10 mg/kg twice a week; N = 13) or an irrelevant isotype matched control IgG specific for Hen egg lysozyme (10 mg/kg twice a week; N = 13). The blocking anti-mouse TWEAK mAb (clone P2D10) was generated in *Tnfsf12*^−/−^ mice^42^. In addition, diabetic mice [*Tnfsf12*^+/+^*ApoE*^−/−^ (N = 12) and *Tnfsf12*^−/−^*ApoE*^−/−^ (N = 12)] and non-diabetic mice [*Tnfsf12*^+/+^*ApoE*^−/−^ (N = 7)] were included to analyze the effect of *Tnfsf12* deletion on diabetic-accelerated atherosclerosis. All mice were maintained under barrier conditions. Water and normal laboratory diet was available *ad libitum*. At the end of the study, 16 h**-**fasted mice were anesthetized (100 mg/kg ketamine and 15 mg/kg xylazine), saline perfused, and killed. Blood samples were collected for biochemistry. Serum total cholesterol, HDL-c, LDL-c and triglycerides were measured in our central laboratory (IIS-Fundación Jiménez Díaz).

### En face of aorta

Atherosclerotic lesions were quantified by en face analysis of the whole aorta and by cross-sectional analysis of the aortic root and the innominate artery. For en face preparations, the aorta was opened longitudinally, from the heart to the iliac arteries, while still attached to the heart and major branching arteries in the body. The aorta (from the heart to the iliac bifurcation) was then removed and was ‘pinned out’ on a white wax surface in a dissecting pan using stainless steel pins 0.2 mm in diameter. After overnight fixation with 4% paraformaldehyde and a rinse in PBS, the aortas were immersed for 6 min in a filtered solution containing 0.5% Sudan IV, 35% ethanol and 50% acetone; and destained in 80% ethanol. The Sudan IV**–**stained aortas were photographed and were used for quantification of atherosclerotic lesions.

### Aortic root and brachiocephalic artery morphometric analysis

Brachiocephalic arteries and hearts containing aortic roots were carefully dissected and frozen in OCT. Aortic roots were sectioned at 5 μm thickness beginning proximally at the first evidence of the aortic valves at their attachment site of aorta. Sections were stained with Oil-red-O/hematoxylin at 100 μm intervals from 0 to 900 μm distal to the site. Maximal lesion area was calculated for each mouse by averaging the values for three sections. The individual maximal lesion areas were further averaged to determine the maximal lesion area for each group. Picrosirius red staining was performed for analysis of collagen content by measuring birefringence to plane-polarized light. Immunohistochemistry was carried out as previously described^20^. Brachiochephalic arteries were serially sectioned in 5 μm thickness from the aortic root to the right subclavian artery. For morphometric analysis, sections of each brachiocephalic artery were stained with modified Russell-Movat pentachrome (Movat) at 90 μm intervals from 0 to 450 μm distal to the aortic root.

### Immunohistochemical analysis

Immunohistochemical analysis was done in all animals included in each group. Primary Abs were the monocyte/macrophage marker CD68 (Ab53444; Abcam), the smooth muscle cell marker smooth muscle actin (Clone 1A4; Sigma), calponin (ab46794; Abcam) and osteopontin (ab216406; Abcam), phospho-STAT1 (33–3400, ThermoFisher Scientific), phospho-STAT3 (sc8019, Santa Cruz Biotechnology) and ICAM-1 (ab124760, Abcam).

Donkey anti-goat biotin, sheep anti-mouse biotin, and goat anti-rat biotin (Amersham) were used as secondary antibodies. ABComplex/HRP was then added and sections were stained with AEC substrate-chromogen (DAKO), counterstained with hematoxylin, and mounted in Pertex (Medite). Incubation without primary Abs and/or irrelevant species- and isotype-matched immunoglobulins was used as a negative control for all immunostainings. For colocalization studies anti-rabbit Alexa Fluor 488 and anti-rat Alexa Fluor 568 were used as secondary Abs.

Computer-assisted morphometric analysis was performed with the Image-Pro Plus software (version 1.0 for Windows). The threshold setting for area measurement was equal for all images. Samples from each animal were examined in a blinded manner. Results were expressed as % positive area versus total area (collagen, macrophages, α-actin, calponin, osteopontin and ICAM-1), or positive nuclei per mm^2^ (p-STAT-1 and p-STAT3). Faltan calponin and osteopontin

### Southwestern histochemistry

This technique was developed to detect *in situ* the distribution and DNA-binding activity of transcriptional factors^11^. Nuclear factor kappa B (NF-**k**B) consensus oligonucleotide from the CCL5 promoter was digoxigenin labelled with a 3**′**-terminal transferase (Roche, Basel, Switzerland). OCT-embedded tissue sections were fixed in 0.5% paraformaldehyde and incubated with 0.1 mg/mL DNase I. The DNA binding reaction was performed by incubation with 50 pmol of the labelled DNA probe in buffer containing 0.25% BSA and 1 μg/mL poly (dI-dC). The sections were then incubated with alkaline phosphatase-conjugated antidigoxigenin Ab, and colorimetric detection was performed as described^11^. Preparations without probe were used as negative controls, and an excess of unlabelled probe was used to test the specificity of the technique. Results are expressed as % positive nuclei per mm^2^.

### Cell Culture

Aortic vascular smooth muscle cells (VSMCs) were isolated from aorta of wild-type mice. Briefly, mice were anesthetized (100 mg/kg ketamine and 15 mg/kg xylazine), saline perfused, and killed. Adhering fat and connective tissue were removed from the thoracic aorta. Vessels were opened longitudinally and preincubated in DMEM (Whitaker) containing 1 mg/mL collagenase (type II, 290 U/mg), penicillin (100 U/mL), streptomycin (100 μg/mL), and glutamine (2 mmol/L) (Sigma) for 15 to 20 minutes at 37**°** C in 95% air/5% CO^2^. Then aortas were minced into 1-mm pieces, incubated for an additional 1.5 to 2 hours, and rinsed twice with PBS to remove the cells, which were counted and seeded at a concentration of 10^4^ cells/cm^2^ in plastic culture flasks (Costar) in DMEM with 10% FBS. Cells were harvested for passaging at 2- to 3-day intervals and used between the second and seventh passages. For experimental analysis, cells were made quiescent by 24-hour incubation in medium without FBS before stimulation with high glucose (35 mmol/L D-Glucose; Sigma-Aldrich) and/or recombinant murine TWEAK (1237-TW; R&D Systems). JAK inhibitor I (sc-204021) was purchased from Santa Cruz Biotechnology.

### Western Blot

Cultured murine VSMCs from different experimental conditions were collected and pelleted. Western blots of cellular proteins were analyzed as previously described^21^. The membranes were blotted with Abs for anti Fn14 antibody (4403; Cell Signaling Technology), phospho-STAT1 (33–3400, ThermoFisher Scientific), phospho-STAT3 (9134, Cell Signaling Technology), and alpha-tubulin (T5168; Sigma).

### RNA extraction and Real-Time PCR

Total RNA from VSMCs or total aorta of mice was obtained by TRIzol method (Life Technologies) and quantified by absorbance at 260 nm in duplicate. Real-time PCR was performed on a TaqMan ABI 7700 Sequence Detection System using heat-activated TaqDNA polymerase (Amplitaq Gold). After an initial hold of 2 minutes at 50**°** C and 10 minutes at 95**°** C, the samples were cycled 40 times at 95**°** C for 15 seconds and 60**°** C for 60 seconds. 18S rRNA served as housekeeping gene and was amplified in parallel with the genes of interest. The expression of target genes was normalized to housekeeping transcripts. The following PCR primers and TaqMan probes were purchased from Applied Biosystems and optimized according to the manufacturer’s protocol: 18S (4310893E), mouse CCL5 (Mm01302427), CXCL10 (Mm00445235), ICAM-1 (Mm00516023), VCAM-1 (Mm01320970) and TNF-α (Mm00443258). All measurements were performed in triplicate. The amount of target mRNA in samples was estimated by the 2**Δ**CT relative quantification method. Values of each sample were obtained as multiples of their baseline values. Mouse mRNA levels for ACTA2 (α-actin), CNN1 (calponin) and Spp1 (osteopontin) were done by amplification of cDNA using SYBR Premix Ex Taq^TM^ (Takara Biotechnology). The primer sequences are: 5′-3 ACTA2 forward: CATCTTTCATTGGGATGGAG, reverse: TTAGCATAGAGATCCTTCCTG; CNN1 forward: AACTTCATGGATGGCCTCAAA, reverse: ACCCGGCTGCAGCTTGT; and Spp1 forward: ATGAGATTGGCAGTGATTTG; reverse: CATCCTTTTCTTCAGAGGAC. The mRNA levels were normalized to the GADPH mRNA content.

### Transfection of Small-Interfering RNA

Murine VSMCs grown to 60**–**70% confluence were transfected with 20**–**30 nmol/L of small interfering RNA (siRNA) targeting Fn14, STAT1 or negative control scramble siRNA (Ambion) using Lipofectamine RNAiMAX reagent (Life Technologies). Transfected cells were treated with rTWEAK (100 ng/mL) in presence of low or high glucose concentrations during 24 hours.

### Statistical analysis

Statistical analysis was performed using SPSS 11.0 statistical software. Values are expressed as mean ± SEM. Differences across groups were considered significant at p < 0.05 using either nonparametric Mann Whitney U test or one-way ANOVA followed by a post hoc Bonferroni pairwise comparison test. *In vitro* experiments were replicated at least 3 times.

## Additional Information

**How to cite this article:** Fernández-Laso, V. *et al*. TWEAK blockade decreases atherosclerotic lesion size and progression through suppression of STAT1 signaling in diabetic mice. *Sci. Rep.* **7**, 46679; doi: 10.1038/srep46679 (2017).

**Publisher's note:** Springer Nature remains neutral with regard to jurisdictional claims in published maps and institutional affiliations.

## Supplementary Material

Supplementary Information
