# Supplementary material for: TWEAK blockade decreases atherosclerotic lesion size and progression through suppression of STAT1 signaling in diabetic mice
Source: Sci Rep. 2017 Apr 27;7:46679. doi: 10.1038/srep46679 (PMC5406837; doi:10.1038/srep46679)
Supplement: Supplementary Information [file srep46679-s1.pdf]

TWEAK blockade decreases atherosclerotic lesion size and progression through suppression of STAT1 signaling in diabetic mice

Valvanera Fernández-Laso, Cristina Sastre, Nerea Méndez-Barbero, Jesús Egido, Jose L. Martín-Ventura, Carmen Gómez-Guerrero, Luis M. Blanco-Colio.

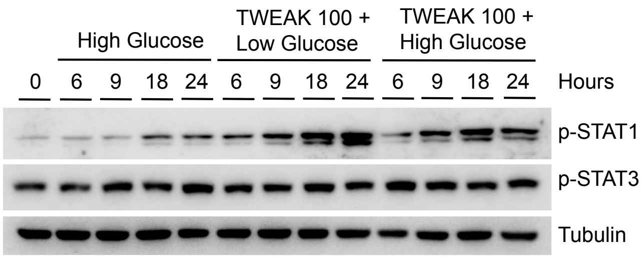

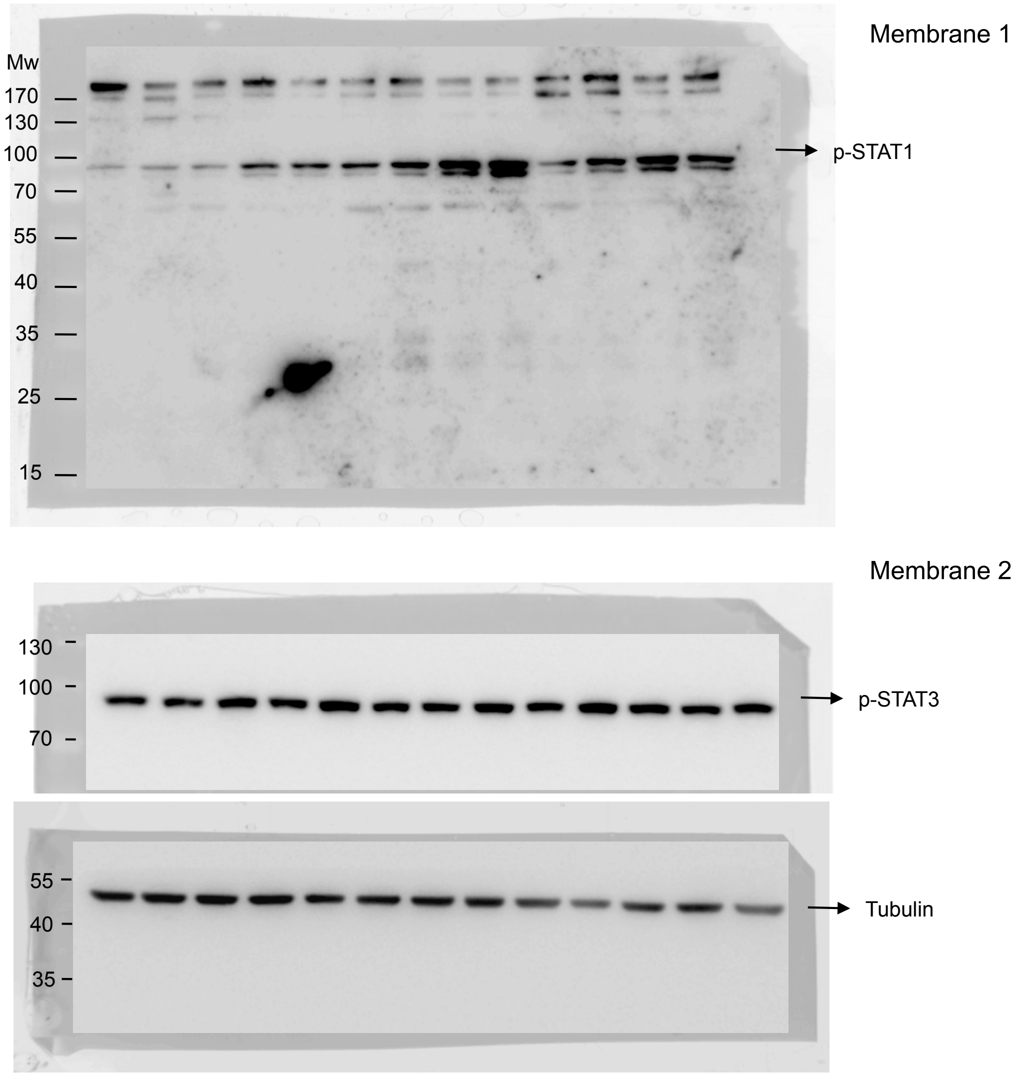

Supplementary Figure S1. Full-image of western-blots shown in figure 4A. Due to the different molecular weights, the second membrane was cropped and then incubated with either anti-pSTAT3 or anti-tubulin as indicated.

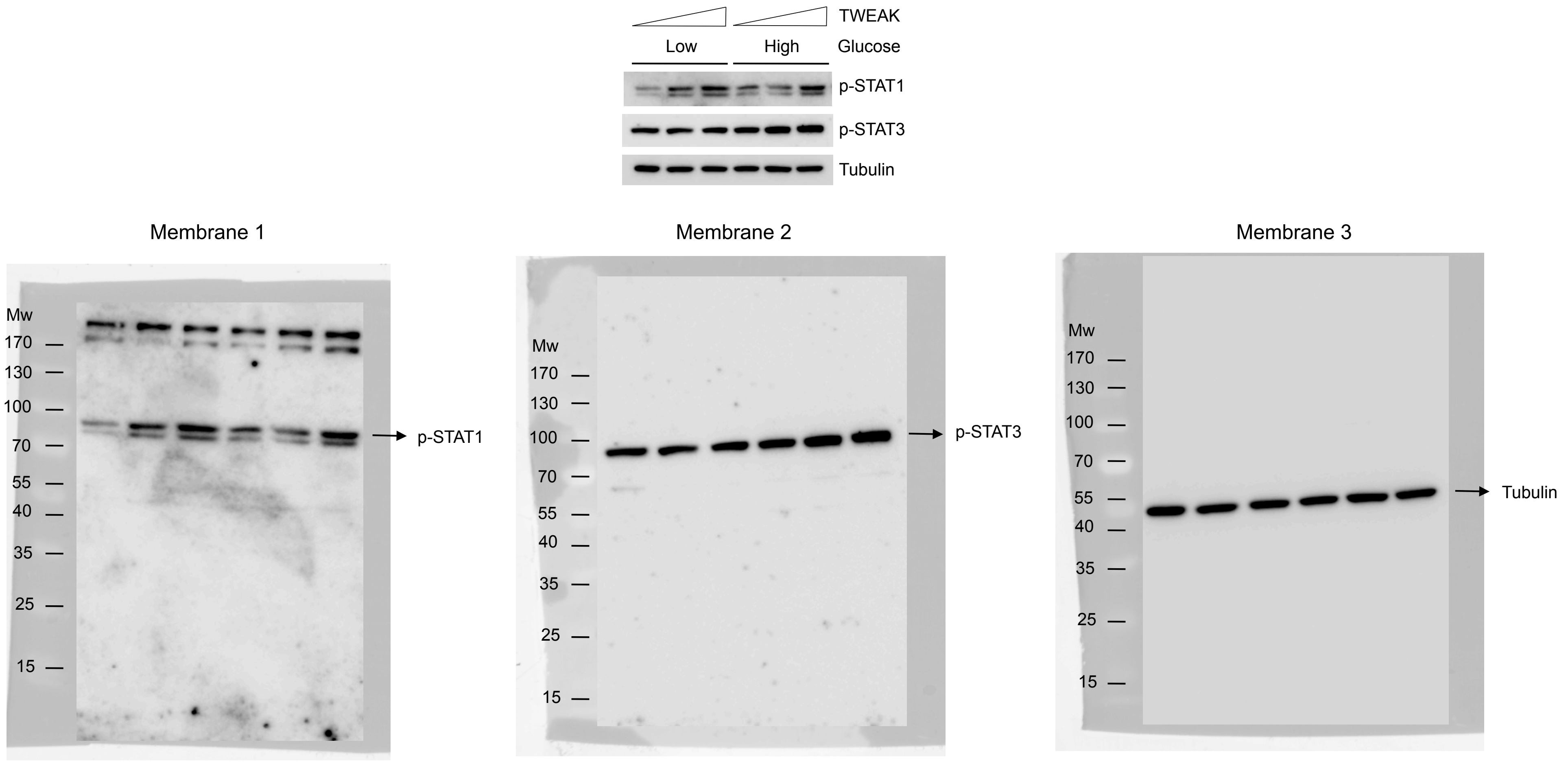

Supplementary Figure S2. Full-image of western-blots shown in figure 4B.

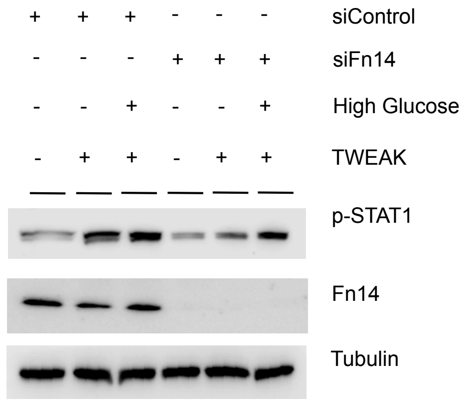

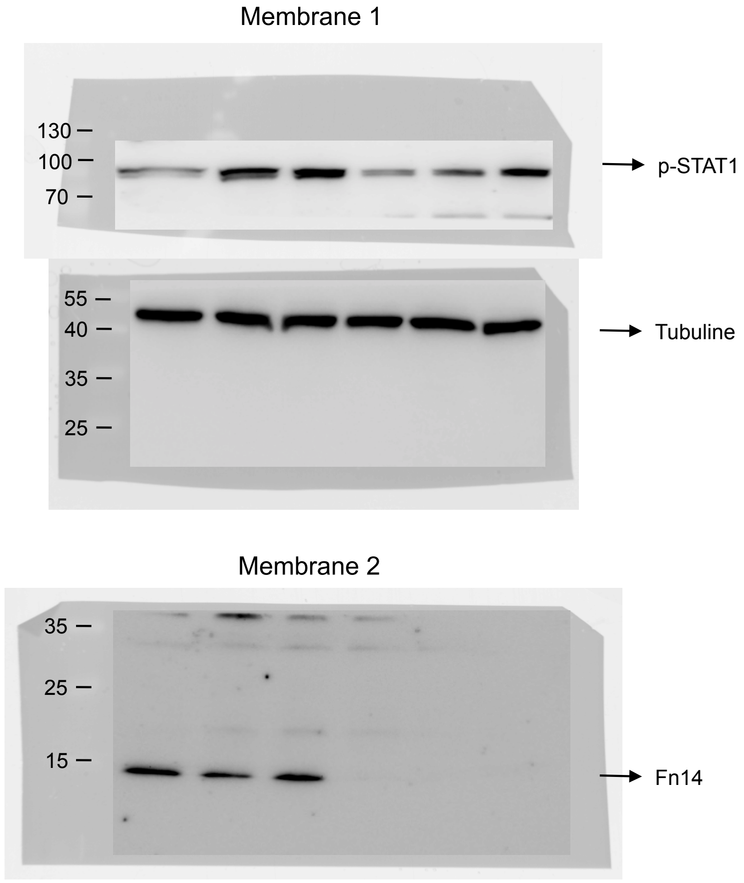

Supplementary Figure S3. Full-image of western-blots shown in figure 4C. Due to the different molecular weights, the first membrane was cropped and then incubated with either anti-pSTAT1 or anti-tubulin as indicated.

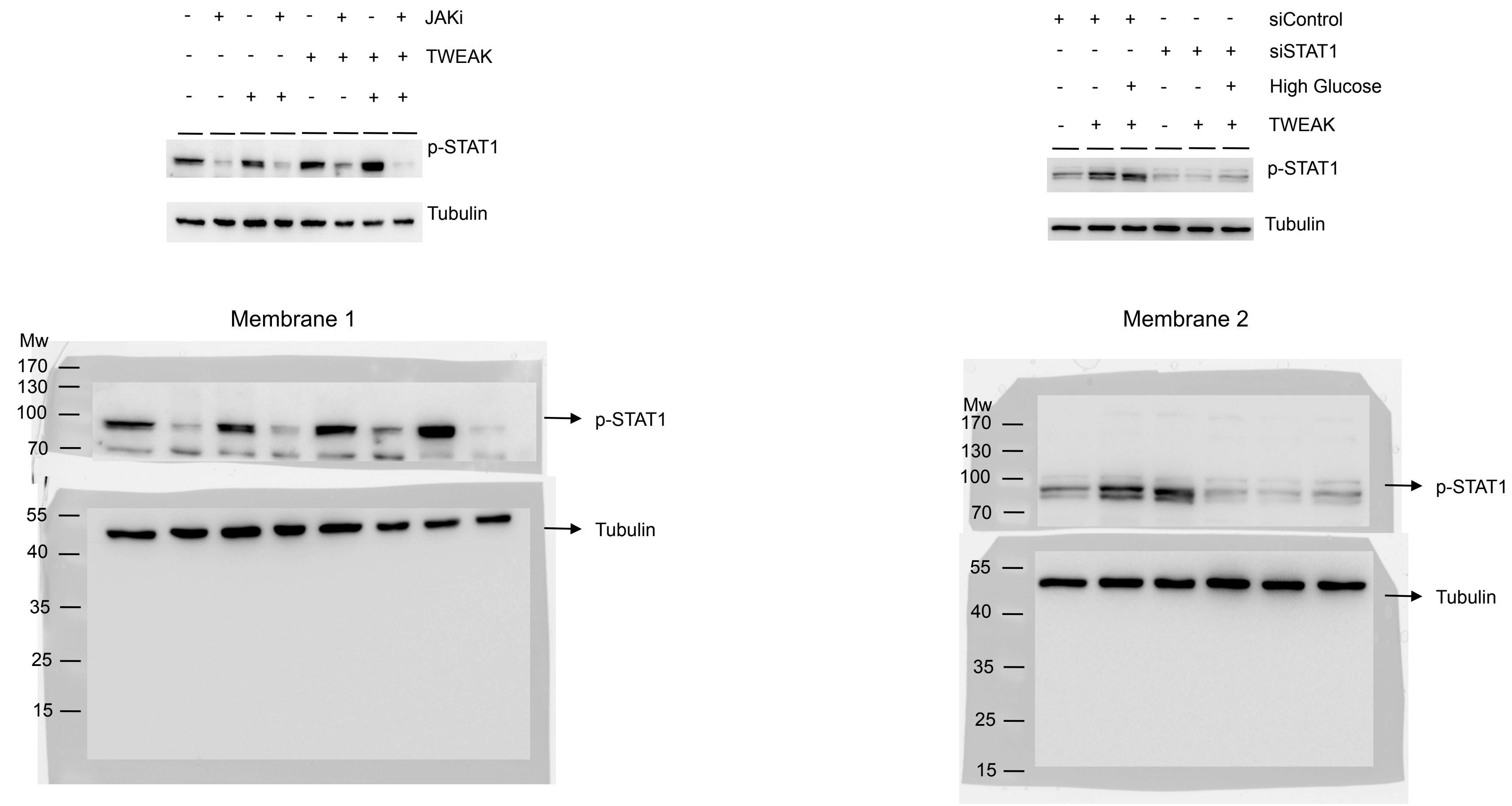

Supplementary Figure S4. Full-image of western-blots shown in figure43D-F. Due to the different molecular weights, the membranes were cropped and then incubated with either anti-pSTAT1 or anti-tubulin as indicated.
